# Influence of Reprocessing on the Properties of PVC-Based Wood–Plastic Composites

**DOI:** 10.3390/polym18121509

**Published:** 2026-06-16

**Authors:** Dario Pervan, Mladen Brezović, Nikola Španić

**Affiliations:** Faculty of Forestry and Wood Technology, University of Zagreb, Svetošimunska Cesta 23, 10000 Zagreb, Croatia; mbrezovic@sumfak.unizg.hr (M.B.); nspanic@sumfak.unizg.hr (N.Š.)

**Keywords:** wood–plastic composite, polyvinyl chloride, mechanical properties, physical properties, reprocessing, thermal characterization, chemical characterization

## Abstract

The reprocessing of wood–plastic composites (WPCs) significantly affects their structural integrity and thermal behavior. Despite this, the effect of reprocessing on PVC-based WPCs has not been extensively investigated, and the mechanism is not well understood. This study evaluated the effect of reprocessing on the properties of a PVC-based WPC. Small pieces of extruded WPC boards (2–4 mesh) were first milled to a granulation of 50 mesh, and then the material was reprocessed by compression molding, with part of the samples reinforced with glass- and carbon-fiber fabric. The physical and mechanical properties of the reprocessed material were analyzed, and the chemical and thermal characteristics of the reprocessed WPC were compared with the virgin WPC. The results of the mechanical and physical property tests showed that the reprocessed WPC had satisfactory properties compared with the virgin WPC. Samples reinforced with carbon-fiber fabric showed a statistically significant increase in tensile and flexural strength in comparison with unreinforced reprocessed WPC samples. Fourier-transform infrared (FTIR) spectroscopy, thermogravimetric analysis (TGA), and differential scanning calorimetry (DSC) showed that partial dehydrochlorination, thermal degradation and a decrease in thermal stability occurred. Overall, the results of this study show that although chemical degradation and a decrease in thermal stability were present in the reprocessed WPC, it retained satisfactory mechanical and physical properties that could be improved by reinforcing it with carbon-fiber fabric.

## 1. Introduction

The global accumulation of plastic waste is one of the most significant challenges of the modern era. This issue arises from the linear economy model, which prioritizes disposal over reuse of materials [1]. The complexity and high cost of recycling plastics, due to the existence of different types and various non-separable additives, result in only 9% of global plastic waste being recycled [2]. This challenge directly highlights the potential of wood–plastic composites (WPCs). These materials enable the valorization of mixed and difficult-to-recycle plastic waste by incorporating it with lignocellulosic components [3].

Wood–plastic composites (WPCs) are polymer materials whose main components are typically thermoplastic materials (most often polyethylene, polypropylene, and polyvinyl chloride) and wood flour or fibers mixed into a matrix. In this matrix, wood most often acts as a filler but can also serve as reinforcement [4]. Since their invention in 1960 in Milan under the name plastic–wood, the production and use of WPCs have expanded considerably. According to market data, the global market for wood–plastic composites reached a volume of 1.2 million tons in 2023, with a value of approximately 6.9 to 7.8 trillion US dollars. Various sources predict an annual growth rate between 7.5% and 13.6% [5]. This significant projected increase is primarily due to the growing use of WPCs in the construction sector and the initial adoption of WPCs in the automotive industry [6]. The main reason for their popularity is their suitability for outdoor use, especially for decking. Compared with highly resistant wood, WPCs are resistant to atmospheric agents, easy to maintain, and offer the possibility of aesthetic adjustment, combined with a lower price [4]. Compared with plastic, they have significantly better properties at higher temperatures and less creep, which allows WPCs to be used for construction purposes [7]. Also, due to the use of wood within WPCs, which can reach up to 50 to 70% of the mass of WPCs, they are also considered a more ecological variant that leaves a smaller environmental impact compared with pure plastic composites [8], although this impact is greater than that of solid wood and other wood composites.

Currently, the most common plastic polymers used in industry are polyethylene (PE), polyvinyl chloride (PVC), and polypropylene (PP), which accounted for 83%, 9%, and 7% of the US market in 2002, respectively. While PE is most commonly used for decking, PVC is most commonly used for doors and window frames, as it offers satisfactory mechanical properties, chemical resistance, and, with the addition of additives, good resistance to UV radiation [9]. After mixing the two raw materials, WPCs are most commonly produced in three ways: extrusion (the most common industrial method), injection molding (for forming complex shapes), and compression molding (more common in laboratory applications). Compared with PE, PVC is more demanding to process due to its rheological properties and sensitivity to heat. When products are made with a large proportion of wood, the problem of under-mixing and release of moisture from the wood can occur, which can lead to uneven density in the final material. To improve the dispersion of wood within the WPC matrix, various lubricants (fatty acids and waxes) are used. Pigments, stabilizers, and plasticizers are also added as needed. Due to the aforementioned problem, PVC-based WPCs are extruded using counter-rotating twin-screw extrusion. Counter-rotating twin-screw extruders are used for heat-sensitive materials such as rigid PVC because they allow processing at lower temperatures, with good mixing, degassing and working with powdery or difficult-to-flow materials. Their advantages are low speed and shear, while the disadvantages are the need for drying, pre-mixing and possible additional material grinding [9,10,11].

Due to the presence of these additives and the inherent properties of PVC, the question arises regarding the feasibility of reprocessing WPCs as a means of recycling damaged or defective WPCs. Previous research on WPC recycling has primarily addressed the use of plastic recyclables (often sourced from waste) as a raw material for WPC production [12]. Although these studies achieved WPCs with satisfactory mechanical properties using the compound-pressing method—particularly when higher proportions of wood were present in the composites [13,14]—most focused on the wood–plastic ratio in composites rather than comparisons with commercial WPCs. The production of a WPC from recycled PE and PP by extrusion resulted in a WPC with inferior mechanical properties and poor interfacial bonding [15].

Reprocessing WPCs as a form of recycling has been much less studied. Reprocessing refers to the reuse of an already defective WPC, most often by crushing the extruded material and reusing it through extrusion or compound pressing. Research on the reprocessing of WPCs based on HDPE showed that the reprocessed material had, on average, 13% lower flexural strength and 14% lower tensile strength compared with virgin WPCs. The decrease in mechanical properties corresponded to the lower density of reprocessed composites compared with virgin composites. The decrease in crystallinity between virgin and recycled WPCs was found not to be significant [16]. In the case of a PP-based WPC, reprocessing up to five times did not show any major degradation apart from color change [12]. It has been shown that adding wood fibers during the reprocessing process can reduce the negative impact of percolation on the mechanical properties of WPCs [17].

Due to their specific processing requirements, research on the reprocessing of PVC-based WPCs is almost non-existent, and most researchers, when dealing with recycled WPCs, imply the use of waste PVC. Therefore, the aim of this study was to investigate the possibilities and properties of a reprocessed WPC based on PVC. Reprocessed WPC boards were made from a shredded, previously damaged WPC. In addition to physical properties and water retention, the obtained samples were chemically and thermally analyzed by Fourier-transform infrared (FTIR) spectroscopy, thermogravimetric analysis (TGA), and differential scanning calorimetry (DSC) in order to identify possible changes in chemical structure, thermal stability and phase transitions compared with the starting material. Because moderate thermal degradation was expected to worsen the mechanical properties [18], some of the samples were reinforced with cladding layers made of glass- or carbon-fiber fabric. The purpose of these layers was to improve mechanical properties, as such synthetic fibers have already proven effective for reinforcing WPCs [19,20]. The results of this work will help us to better understand the effects of reprocessing on PVC-based WPCs. This includes changes in their chemical composition and mechanical and structural properties. In addition, this study will provide insight into the potential for reinforcing such materials and improving their performance for broader engineering and construction applications.

## 2. Materials and Methods

### 2.1. Materials

The samples for the production of test panels were obtained from extruded Twinson P 9360 PVC-based WPC boards (Deceuninck, Hooglede-Gits, Belgium). The reinforcing fabrics used in this work were Interglas UTE 163 g/m^2^ glass-fiber fabric (Faserverbundwerkstoffe, Waldenbuch, Germany) with a twill weave and HinFab TM HCP200C carbon-fiber fabric (Hindostan Technical Fabrics, Mumbai, India) with a fiber diameter of 7 µm and a weave thickness of 0.2 mm.

### 2.2. Sample Preparation

**Figure 1:**
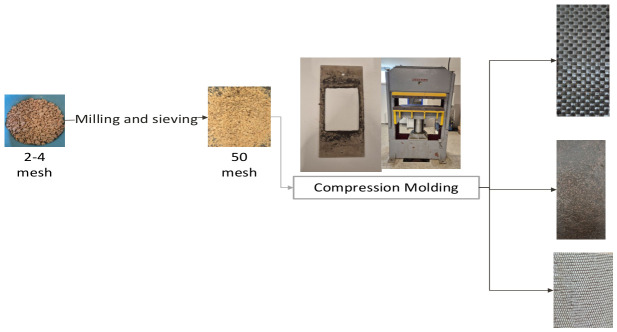
Schematic diagram of the reprocessing procedure.

The WPC material was supplied in the form of small pieces (2–4 mesh) and was subsequently milled using a Retsch ZM 200 mill (Retsch GmbH, Haan, Germany). The resulting material was sieved to a particle size of 50 mesh, which is within the appropriate range for achieving optimal properties for a pressed WPC [21,22,23]. The milled sample was then placed in a metal mold. The mold had internal dimensions of 160 mm in length, 100 mm in width, and 3 mm in thickness. A Belišće industrial press (Belišće d.d., Belišće, Croatia) was used for the production of the composites. It was found that 60 g of sample provided the best mold filling and the most uniform composite thickness, so the samples were produced using this amount. The production process followed the following parameters: preheating to 170 °C for 8 min, and then pressing at a pressure of 0.5 N/mm^2^ and a temperature of 170 °C for 15 min. After hot pressing, the samples were placed in a cold press for 5 min at a pressure of 5 N/mm^2^ to maintain their shape. For samples coated with glass- or carbon-fiber cloth, the fabric was placed on the bottom and top of the mold before the sample was placed in the hot press, so that during pressing, the molten polymer penetrated into the pores and between the fibrous spaces of the fabric, resulting in mechanical interlocking during cooling. A schematic diagram of the reprocessing procedure is shown in Figure 1.

### 2.3. Determination of Density, Moisture Content and Porosity of WPC Matrix

Density was determined according to ASTM D 792-07 [24]. Samples with a mass of approximately 1.5 g were weighed in air and then weighed while submerged in water using a wire or a sinker, and the specific gravity and density were then calculated. The moisture content of the WPC matrix was determined according to ASTM D 4442-92 [25], using method A (primary oven-dry method). Porosity was determined using the method described by Wang et al. [26]. This method was modified to better suit the WPC, as shown in the following Equation (1):

Porosity = ((W_a_ − W_b_)/Ρ_w_)/(W_b_/Ρ_c_) (1)

where W_a_ is the weight of the WPC after being soaked in water, W_b_ is the weight of the dry and conditioned WPC, and Ρ_w_ and Ρ_c_ are the density of water and the WPC.

For each mentioned test, a minimum of 3 samples were used.

### 2.4. Chemical and Thermal Characterization

The infrared spectrum of the virgin WPC and the reprocessed WPC was recorded on a Shimadzu Fourier-infrared spectrophotometer (FTIR) 8400S (Shimadzu, Kyoto, Japan) with the IRsolution software (Shimadzu Corporation, Kyoto, Japan) (v1.60) to determine possible changes in chemical structure and functional groups. FTIR spectra were obtained in transmission mode using the KBr pellet method with a spectral resolution of 4 cm^−1^ across the 4000–400 cm^−1^ range. The pellets (13 mm in diameter) were prepared by thoroughly mixing 10 mg of the sample with 300 mg of dry, spectroscopic-grade potassium bromide (KBr), followed by compression for 5 min at 200 bar using a PIKE press equipped with a die set. Each sample was measured twice, with 10 scans recorded per measurement, resulting in spectra based on an average of 20 scans in total.

Thermal characterization of the virgin and reprocessed WPCs was conducted using a TGA 4000 thermo-gravimetric analyzer (PerkinElmer, Waltham MA, USA) and a differential scanning calorimeter DSC 6000 (PerkinElmer, Waltham, MA, USA) with the PYRIS (PerkinElmer) (v14) software. Thermogravimetric analysis was performed with approximately 13 mg of the sample, heated from 50 to 770 °C at a rate of 10 °C/min in a compressed air atmosphere. The purge rate was set at 25 mL/min, and two replicates were prepared per sample. Differential scanning calorimetry was carried out according to the procedure described in a previous study [27] to determine the change in the glass transition temperature after reprocessing. About 6 mg of the crushed sample was sealed in an aluminum container and placed in the device under a nitrogen atmosphere. The samples were first heated from 25 to 160 °C and then cooled from 160 to 25 °C to remove the thermal history of the material. They were then reheated from 25 to 160 °C, and the resulting thermogram was used for further analysis. The heating and cooling rates were 10 °C/min. Two replicates were performed per sample.

### 2.5. Determination of Water Absorption

Water absorption was measured according to ASTM D 570-98 [28]. Three specimens were taken from each group (the reprocessed WPC, the reprocessed WPC reinforced with glass-fiber fabric, and the reprocessed WPC reinforced with carbon-fiber fabric). The test followed procedures 7.1 and 7.5. According to procedure 7.1 of the standard, the specimens were immersed in distilled water at 23 ± 1 °C for 24 h, and then their surfaces were wiped with a dry cloth and weighed. Specimens tested according to procedure 7.5 were immersed in boiling water for 120 ± 1 min, and then removed, wiped with a cloth, and weighed. Following this, the samples were reconditioned in order to determine the value of the soluble fraction (soluble matter lost). The results were calculated as the percentage mass increase after immersion and the amount of soluble matter lost during immersion, as shown in the following Equations (2) and (3):

Increase in weight = (m_w_ − m_c_)/m_c_ (2)

Soluble matter lost = (m_c_ − m_rc_)/m_c_ (3)

where m_w_ is the weight of the WPC after being soaked in water, m_c_ is the weight of the conditioned WPC, and m_rc_ is the weight of the reconditioned specimen after it was soaked in water.

For each mentioned test, a minimum of 3 samples were used.

### 2.6. Determination of Mechanical Properties

Tensile properties were determined according to the ASTM D 3039 standard [29] using an AG-X plus (Shimadzu, Kyoto, Japan) testing machine with a nominal force of 20 kN. The sample dimensions were adapted to the standard, except for the length (max. length of 160 mm due to mold limitations). The final sample dimensions were 160 mm × 25 mm × 3 mm (length × width × thickness). The tensile test speed was set at 2 mm/min. Bending properties were determined according to the ASTM D 7264 standard [30] for three-point bending using the Schenck Trebel UPM 20 T (Schenck Trebel, New York, NY, USA) testing machine with a nominal force of 10 kN at a speed of 3 mm/min. The distance between supports was 110 mm. The sample dimensions were 115 mm × 13 mm × 3 mm (length × width × thickness). Tests were conducted at 23 ± 2 °C and 50 ± 10% RH. At least five samples per group were used for each mechanical property test. Flexural properties were calculated using Equations (4) and (5):

σ_f_ = (3 × P_max_ × L)/(2 × b × d^2^) (4)

E_f_ = (m × L^3^)/(4 × b × d^3^) (5)

where P_max_ is the maximum load (N), L is the support span (mm), b and d are the sample width and thickness (mm) and m is the slope of the load–deflection curve in the linear region (N/mm).

### 2.7. Statistical Analysis

SPSS Statistics (IBM) (v2025) and Excel (Microsoft) (v2019) were used for statistical data analysis. For the statistical evaluation of the experimental data, the Kruskal–Wallis test was used to determine whether significant differences existed between the sample groups. This nonparametric method was selected due to the smaller sample size and the potential deviation from a normal distribution.

## 3. Results and Discussion

### 3.1. Physical Properties

The results of the density, moisture content and porosity of the reprocessed WPC are presented in Table 1.

The average density of the reprocessed samples was 1289.61 kg/m^3^, with minor variations between samples. This value is consistent with standard values for virgin WPCs based on PVC (1000–1300 kg/m^3^) [9]. The absence of a significant density reduction after reprocessing indicates that the hot-pressing cycle did not cause substantial internal void formation. This contrasts with other types of WPC, where reprocessing by extrusion or injection molding resulted in decreased density [16]. The moisture content of the reprocessed composite was also within the typical range for WPCs with lower wood fiber contents [31] but, on average, slightly higher than that for WPCs based on HDPE [32]. The low moisture content of WPCs in general can be attributed to the encapsulation of wood particles within the hydrophobic PVC matrix, which limits moisture sorption and reduces the accessibility of hydrophilic hydroxyl groups [31]. The porosity was at the lower end of the range for WPCs, indicating that the matrix material was well melted and bonded during pressing. Generally, porosity values are lower during press forming due to the application of increased pressure, which improves material compaction and reduces the amount of trapped air, thereby minimizing the formation of internal voids and microdefects in the composite structure [33].

### 3.2. FTIR, TGA and DSC Results

**Figure 2:**
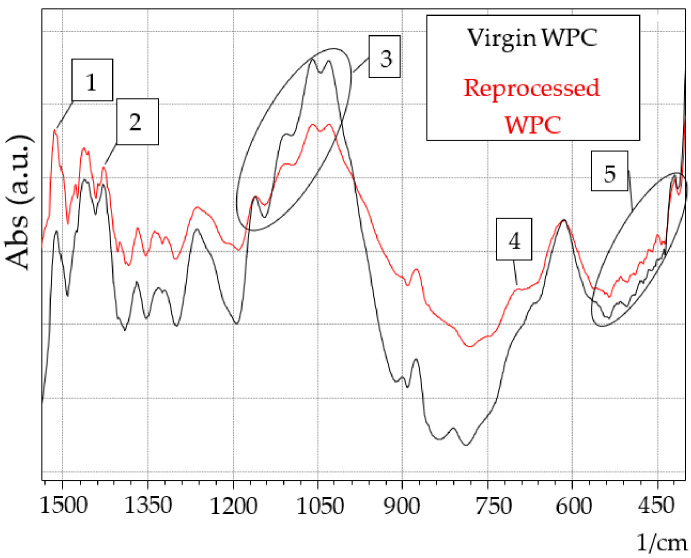
FTIR spectra for virgin and reprocessed WPCs in the range of 1550–300 cm^−1^.

**Figure 3:**
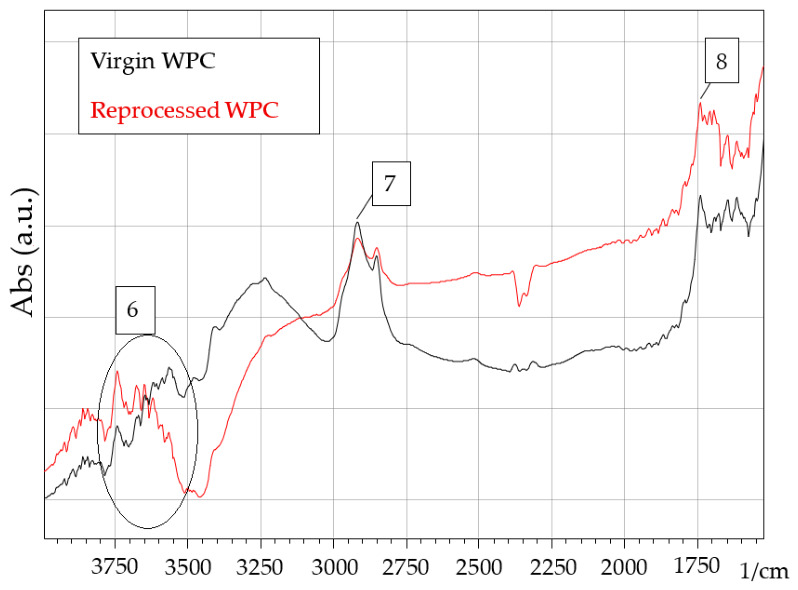
FTIR spectra for virgin and reprocessed WPCs in the range of 4000 to 1500 cm^−1^.

The Fourier-transform infrared spectroscopy (FTIR) results are presented in Figure 2 and Figure 3.

With the reprocessed WPC, the appearance of the initial stage of thermal degradation of the PVC matrix and the lignocellulosic component was visible. In the region below 600 cm^−1^ for PVC, characteristic C–Cl stretching bands (418–558 cm^−1^) [34] were reduced both in number and intensity (peaks in region 5). This change indicates that a partial dechlorination of PVC had occurred. Meanwhile, a new band at 693 cm^−1^ (peaks in region 4) appeared in the spectrum of the reprocessed WPC sample, which could be overwritten by C-H bond deformations in conjugated polyene sequences (–CH=CH–)ₙ as a result of elimination of HCL, although overlap with residual C–Cl vibrations cannot be excluded [35]. Modification of the PVC matrix was further confirmed by a reduction of more than 50% in the band areas at 1427 cm^−1^ (region 2, CH_2_ scissoring) and 2917 cm^−1^ (region 7, asymmetric C–H stretching), suggesting chain scission and degradation of aliphatic structures in the PVC matrix [36]. Changes were also observed in the lignocellulosic fraction. The band at 1510 cm^−1^ (peaks in region 1) was assigned to aromatic skeletal vibrations (C=C) of syringyl units (lignin) and decreased in area by 75%. Suggesting substantial modification and partial degradation of the lignin structures. Simultaneously, the band at 1510 cm^−1^ became broader and less pronounced, suggesting accumulation of degraded phenolic products [37]. The bands characteristic of C–O and C–O–C linkages in carbohydrates (C_6_H_10_O_5_)_n_, located at 1030, 1058, 1103, and 1158 cm^−1^ (peaks in region 3), recorded area reductions of 46% to 60% after reprocessing. Particularly significant was the loss of the band at 1030 cm^−1^ (C-O stretching of the C-6 and C-3 hydroxyl groups) from 2.51 to 1.01, indicating partial thermal modification of cellulose chains [38]. Hemicellulose, being more amorphous and thermally less stable, appeared to be completely degraded, as evidenced by the almost total disappearance of sharp OH bands above 3500 cm^−1^, characteristic of crystalline cellulose domains [39]. Although oxidation was expected during reprocessing, there was only a small increase in carbonyl species. The band at 1729 cm^−1^ (peaks in region 8) increased from 0.049 to 0.10, while a weak new band appeared at 1768 cm^−1^ (peaks in region 8), possibly indicating the formation of additional oxidized carbonyl structures [40]. However, the band at 1739 cm^−1^ (peaks in region 8) (likely an ester from additives or a coupling agent) remained almost unchanged. The relatively low level of oxidation suggests that it plays a secondary role compared with thermal degradation (dehydrochlorination and wood degradation) [41]. One of the most noticeable differences between the virgin and reprocessed WPCs was the complete loss of the crystalline order of cellulose. The virgin WPC contained a series of sharp, well-resolved bands in this region (3410, 3478, 3527–3589, 3608–3677, 3714, 3732, and 3745 cm^−1^) (peaks in region 6), characteristic of crystalline cellulose domains or OH groups on the surface of hydroxylated fillers (e.g., talc or kaolin). After reprocessing, this characteristic pattern almost completely disappeared, with only very weak bands remaining at 3565–3678 cm^−1^ with areas below 0.78 [38,42]. All of this is a strong indication that during the reprocessing of the WPC, dechlorination of the PVC component and deterioration of the lignocellulosic matrix primarily occurred, while oxidation remained limited.

**Figure 4:**
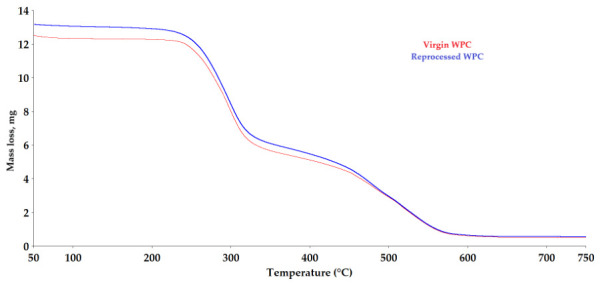
Thermogram of virgin WPC (red) and reprocessed WPC (blue).

**Figure 5:**
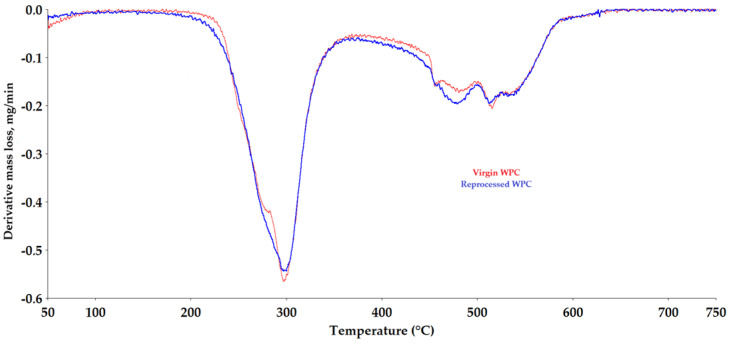
DTG thermograms of virgin WPC (red) and reprocessed WPC (blue).

The thermogravimetric (TG) behavior of both materials (Figure 4 and Figure 5), together with the DTG results presented in Table 2, showed a similar three-step degradation pattern. The first and largest stage of mass loss can be attributed to the dehydrochlorination of the PVC component of the matrix [43], overlapping with the lignocellulosic component. The onset temperature of the virgin WPC (258.13 °C) was higher than that of the reprocessed WPC (217.84 °C), confirming the better thermal stability of the virgin samples. Although degradation began earlier in the reprocessed material, the degradation mechanism remained the same, as indicated by the degradation peak occurring at nearly the same temperature (296.89 °C for the virgin samples and 297.63 °C for the reprocessed samples). A similar magnitude of mass loss was observed during the first stage of degradation. This behavior is commonly associated with chain scission, partial stabilizer depletion, and accelerated degradation due to previous thermal exposure from reprocessing [44]. The second degradation event can be attributed to the decomposition of thermally more stable structures formed during the first degradation. For PVC, this involves cyclization and fragmentation of the resulting polyene sequences [35,36]; for the wood component, it involves the decomposition of lignin-derived char. Unlike cellulose and hemicellulose, lignin degrades over a broad temperature range and contributes significantly to char formation [45]. In the reprocessed WPC, the second degradation event shifted to higher temperatures. This suggests that the reprocessed material contained a larger fraction of thermally resistant secondary residue formed during the first cycle. A higher mass loss indicated more pronounced decomposition of the reprocessed material in this degradation phase. The third degradation phase was also shifted to a higher onset temperature, but T_max_ remained similar. This stage of degradation represents carbonaceous residues remaining after the previous degradation events (condensed aromatic structures, graphite-like carbon residues, highly carbonized lignin char, and residual PVC-derived carbonaceous material). Leftover residue decomposes slowly at high temperatures due to its high thermal stability [35,36,45]. Similar values of T_max_ indicate that the structure of that char remained comparable in both WPCs. The total mass loss during this analysis was 95.83% for the reprocessed WPC and 95.94% for the virgin WPC.

This method of decomposition corresponds to the overlapping degradation of wood and PVC. The initial decomposition of wood starts at 200–260 °C due to the decomposition of hemicellulose. The main degradation peak occurs at 320–370 °C, which represents cellulose degradation. Lignin decomposes over a wide range of temperatures extending up to 600 °C. In comparison, PVC undergoes two-stage degradation: first, dehydrochlorination at temperatures from 250 to 350 °C, and second, decomposition of polyene sequences at temperatures above 400 °C. According to these results, it is evident that reprocessing primarily affects the initial thermal stability, while the degradation mechanism itself does not change significantly [35,36,41,45].

The results of DSC (differential scanning calorimetry) for determination of the glass transition temperature (T_g_) are presented in Table 3.

DSC showed a decrease in the T_g_ during reprocessing, from 75.84 °C for the virgin WPC to 71.97 °C for the reprocessed WPC. These values are lower compared with commercial PVC and PVC-based WPCs, which are reported in the literature to range between 79 and 90 °C. A reduction in the Tonset and Tendset values was also recorded, confirming the decrease in the thermal stability of the reprocessed material. This also indicates that less thermal energy was required to initiate segmental motion in the polymer chains. An increase in the heat capacity change (ΔCp) was observed after reprocessing, rising from 0.092 J/g°C to 0.146 J/g°C. This likely indicates reduced structural organization within the composite and an increase in the amorphous fraction [27,46]. These values confirm and correlate with the results of the FTIR analysis, which showed PVC dehydrochlorination, loss of cellulose crystallinity, and degradation of ordered lignocellulosic domains, as well as the TG analysis, which showed greater thermal degradation of the reprocessed WPC compared with the virgin WPC.

### 3.3. Mechanical Properties

**Figure 6:**
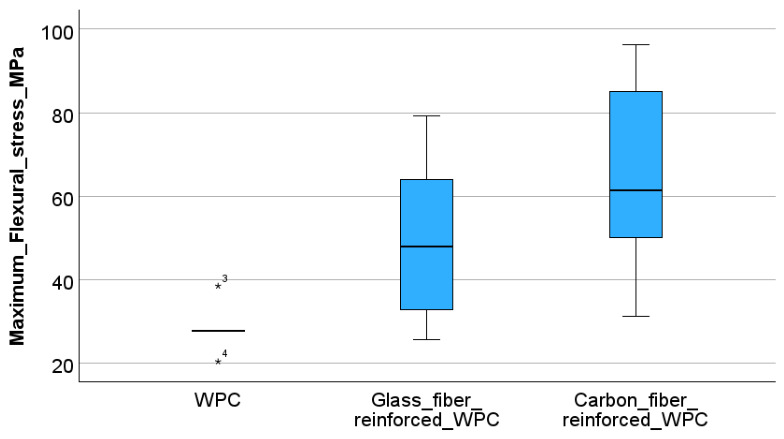
Flexural strengths of reprocessed and fiber-reinforced reprocessed WPCs.

**Figure 7:**
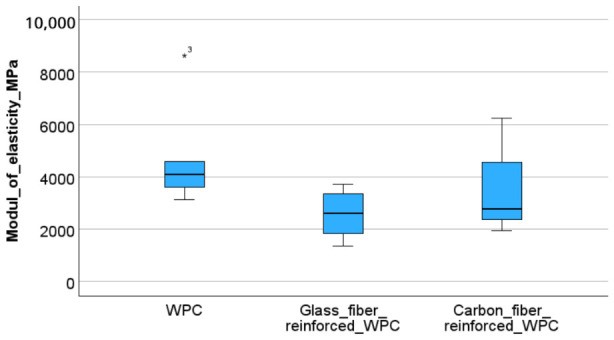
Flexural moduli of elasticity of reprocessed and fiber-reinforced reprocessed WPCs.

Figure 6 and Figure 7 show the average values of the flexural strength and the flexural modulus of elasticity.

**Figure 8:**
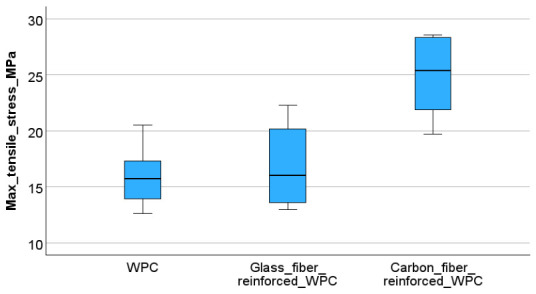
Tensile strengths of reprocessed and fiber-reinforced reprocessed WPCs.

The results generally showed that glass- and carbon-fiber reinforcements increased the flexural strength values (49.31 and 66.74 MPa) compared with the uncoated reprocessed WPC (28.45 MPa). The results confirm the trend of fiber reinforcement in WPCs [47]. The pronounced deviations observed in the flexural strength of the reinforced composites are probably due to poorer adhesion and penetration of the matrix into the fabric during the pressing process. Poorer adhesion was particularly evident in the composites coated with glass fibers, as also reported in previous works based on reinforced extruded WPCs [47]. Although fiber reinforcement resulted in higher flexural strength values, the elastic modulus values of the reinforced composites (2575 MPa for glass fibers and 3430.83 MPa for carbon fibers) were, on average, lower than those of the unreinforced recycled composite (4802.61 MPa). Since the reinforced samples could be considered as sandwich structures, the higher modulus of elasticity exhibited by the unreinforced sample compared with the reinforced samples is mainly attributed to the fact that the elasticity of the system was largely defined by the rigid WPC core. The incorporation of the fiber surfaces into the composite created some interfacial inconsistency, which led to a certain degree of tolerance. Given the nature of the reinforcements used, these fibers were more effective in providing fracture resistance under high-load conditions rather than in increasing stiffness in the early stages. In addition, there may have been some imperfections in the consolidation process between the core and the fibers. A similar trend was also visible for the tensile strength (Figure 8). The carbon-fiber-reinforced samples showed the highest tensile strength value on average (24.78 MPa). The tensile strength value of the unreinforced samples was 15.86 MPa, and that of the samples reinforced with glass fibers was 16.98 MPa. The glass-fiber reinforcement also produced the highest variability, indicating poor stress transfer from the WPC matrix to the fiber, probably caused by incomplete adhesion between the matrix and the fiber during compression. The samples reinforced with carbon fibers showed much better stress transfer. The compatibility and penetration of the WPC matrix during compression were better in relation to the carbon fibers. Carbon fibers are typically supplied with polymer-compatible sizing that enhances wetting and interfacial adhesion during hot pressing, resulting in more efficient stress transfer. In contrast, glass fibers are often optimized for thermoset systems, so their sizing is less compatible with the softened WPC matrix. The obtained mechanical properties of the reprocessed WPC were within the range previously reported in the literature for laboratory-made WPCs [22,48,49,50,51], while the tensile strength was higher, on average, than previously reported [46].

The Kruskal–Wallis test was performed to see if there were statistically significant differences between the samples. The results are shown in Table 4. As might be expected from the results of the mechanical properties of the reinforced WPC, they did not show a statistically significant effect on the elastic modulus, while they were statistically significant for the flexural and tensile strengths. Bonferroni-adjusted pairwise comparisons revealed that the carbon-fiber-reinforced WPC had significantly higher tensile and flexural strength compared with the unreinforced WPC. Glass-fiber reinforcement did not produce statistically significant improvements.

### 3.4. Water Absorption

**Figure 9:**
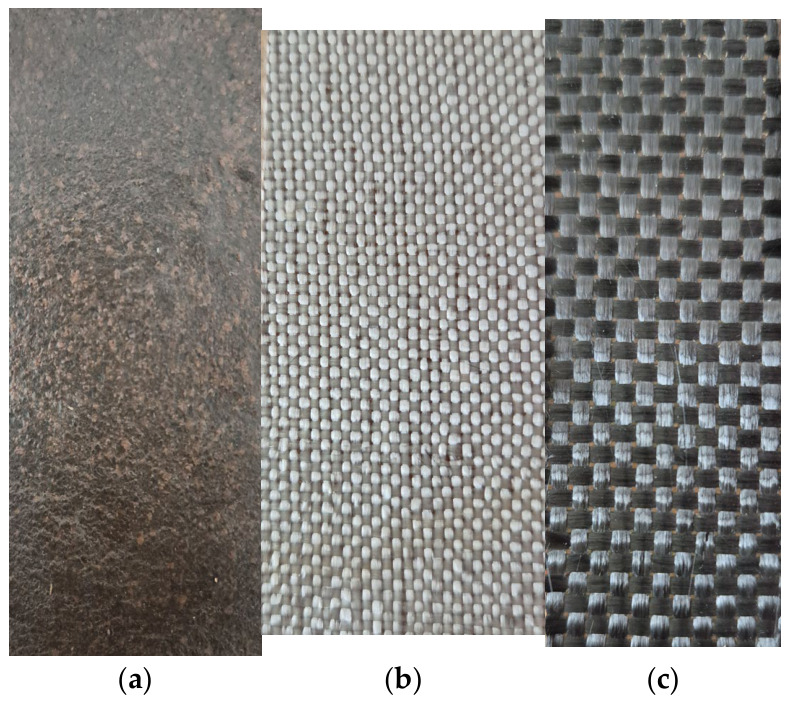
Surface morphology of WPC showing (**a**) unreinforced reprocessed WPC matrix; (**b**) glass-fiber fabric distribution and WPC matrix coverage; and (**c**) carbon-fiber fabric distribution and WPC matrix coverage.

The water absorption results are shown in Table 5. According to the results shown, it is evident that the absorption depended on the type of reinforcement and the test itself. The samples coated with glass fibers showed the lowest water absorption and soluble matter loss in both tests. The unreinforced samples and the samples reinforced with carbon fibers showed, on average, higher values of both soluble matter loss and water absorption in both tests. The absorption values of the reprocessed WPC were significantly higher compared with the values for the virgin WPC [52] but corresponded to the values for recycled WPCs [13]. Enhanced exposure of the hydrophilic wood phase during reprocessing and the inevitable occurrence of microdefects contributed to increased water uptake [16]. Although individual glass and carbon fibers are inherently hydrophobic due to their low surface energy and, in the case of glass, non-porosity, carbon-fiber fabrics are typically coated with polymer-compatible sizing, which increases the water absorption of the fiber cloth [53,54]. Also, due to the weaving method used, glass-fiber fabric provides a better surface coverage than carbon-fiber fabric (Figure 9).

## 4. Conclusions

Overall, this study demonstrated that PVC-based wood–plastic composites (WPCs) can be successfully reprocessed by compression molding. The mechanical properties of the reprocessed WPCs were comparable to those of commercial WPCs. The physical properties, such as density and porosity, were also within the range reported for commercial PVC-based WPCs, highlighting the suitability of compression molding as a reprocessing approach. Water absorption was the only physical property that was, on average, higher than that of standard commercial PVC-based WPCs, which can be attributed to the increased exposure of the lignocellulosic component resulting from milling prior to compression molding. The reprocessed WPC samples reinforced with carbon-fiber fabric exhibited the most effective reinforcement, as the carbon-fiber-reinforced composites demonstrated higher flexural and tensile strengths compared with both the glass-fiber-reinforced and unreinforced reprocessed WPCs. The glass-fiber-reinforced reprocessed WPC samples exhibited only marginal improvements in mechanical properties, and the observed differences from the unreinforced reprocessed WPC samples were not statistically significant. Chemical and thermal analyses showed signs of material degradation after reprocessing; however, the mechanical and physical properties remained within acceptable ranges. This indicates that additional reprocessing cycles may start to affect the physical and mechanical properties of reprocessed WPCs. Future research should investigate the effect of multiple reprocessing cycles on WPCs to determine the recycling limit before mechanical performance becomes unsatisfactory, as well as the use of additives to mitigate thermal degradation during WPC reprocessing.

## Figures and Tables

**Table 1:** Density, moisture content, and porosity of reprocessed WPC.

Variable	N	Min.	Max.	Range	Mean	Median	CV (%)
Density (kg/m^3^)	3	1250.74	1333.76	83.02	1289.61	1284.33	3.30
Moisture content (%)	3	2.8429	3.0046	0.1617	2.9007	2.8546	3.19
Porosity (%)	3	1.71	3.18	1.47	2.31	2.05	33.3

N—number of samples used; CV—coefficient of variation.

**Table 2:** Main thermogravimetric degradation stages of virgin and reprocessed PVC-based WPC samples obtained from DTG analysis.

Stage	Sample	T,_onset_ (°C)	T,_max_ (°C)	Mass Loss (mg)
1	Virgin WPC	258.13	296.89	−5.6345
1	Reprocessed WPC	217.84	297.63	−5.5815
2	Virgin WPC	451.28	455.39	−0.2505
2	Reprocessed WPC	461.23	474.86	−0.3636
3	Virgin WPC	505.21	539.56	−0.7394
3	Reprocessed WPC	525.74	538.79	−0.7298

**Table 3:** DSC parameters of virgin and reprocessed PVC-based WPCs.

Sample	T,_onset_ (°C)	T,_g_ (°C)	T,_endset_ (°C)	ΔCp (J/g·°C)
Virgin WPC	73.30	75.84	78.27	0.092
Reprocessed WPC	68.93	71.97	75.20	0.146

ΔCp—heat capacity change.

**Table 4:** Kruskal–Wallis and Bonferroni-adjusted post hoc comparison results for mechanical properties.

Property	H	df	*p*-Value	Significant Pairwise Differences (Adjusted *p*)
Tensile strength	9.546	2	0.008	WPC vs. carbon-fiber-reinforced WPC (0.010)
Flexural strength	10.388	2	0.006	WPC vs. carbon-fiber-reinforced WPC (0.004)
Flexural modulus	5.622	2	0.060	None

H—test statistic; df—degrees of freedom.

**Table 5:** Water absorption and soluble matter loss values of WPC composites under different immersion conditions (mean ± SD; n = 3).

Sample	24 h Immersion Water Uptake (%)	24 h Soluble Matter Loss (%)	2 h Boiling Water Uptake (%)	2 h Boiling Soluble Matter Loss (%)
WPC	11.27 ± 2.43	2.30 ± 1.04	23.21 ± 8.93	1.73 ± 2.26
Glass-fiber-reinforced WPC	9.53 ± 2.36	0.88 ± 0.38	12.29 ± 1.81	0.05 ± 0.08
Carbon-fiber-reinforced WPC	14.26 ± 2.06	2.48 ± 0.47	30.67 ± 1.99	0.63 ± 0.68

## Data Availability

The original contributions presented in this study are included in the article. Further inquiries can be directed to the corresponding author.
